# Combining biotechnology with sustainability: feasibility of bioenergy sorghum in generating high-value bioproducts

**DOI:** 10.1007/s10529-025-03623-2

**Published:** 2025-08-01

**Authors:** Trine B. Andersen, Elliot Braun, Brianna N. I. Brown, Bjoern Hamberger, Leah Knoor, İlayda Korkmaz, Lucas Reist, Luke Sharpe, Brian Adam McKinley, James O. Suggitt, Mitchell A. Ticoras, Angel Indibi, Sorghum with enhanced economic traits (SWEET) consortium, Trine B. Andersen, Brian Adam McKinley

**Affiliations:** 1 Molecular Plant Sciences Program, Michigan State University, East Lansing, Michigan 48824 USA; 2 Plant Resilience Institute, Michigan State University, East Lansing, Michigan 48824 USA; 3 Genetics and Genome Sciences Program, Michigan State University, East Lansing, Michigan 48824 USA; 4 Department of Plant Biology, Michigan State University, East Lansing, Michigan 48824 USA; 5 DOE Great Lakes Bioenergy Research Center, Michigan State University, East Lansing, Michigan 48824 USA; 6 Department of Biochemistry and Molecular Biology, Michigan State University, East Lansing, Michigan 48824 USA; 7 Department of Biochemistry & Biophysics, Texas A&M University, College Station, TX USA; 8 Department of Microbiology, Genetics, and Immunology, Michigan State University, East Lansing, Michigan 48824 USA; 9 DOE Plant Research Laboratory, Michigan State University, East Lansing, Michigan 48824 USA; 10 Cell and Molecular Biology Program, Michigan State University, East Lansing, Michigan 48824 USA

**Keywords:** Bioenergy sorghum, Machine learning, Societal impact, Technoeconomic analysis, Terpenoid

## Abstract

Biofuels generated through fermentation of plant-derived lignocellulosic biomass are currently not competitive with fossil fuels. Here, a framework is described for the engineering of valuable coproducts to improve the economic feasibility of biofuel production. To accomplish this goal, the desirable traits of the bioenergy crop *Sorghum bicolor* are harnessed via modern engineering approaches. A robust analysis is provided to highlight the scientific basis, costs, regulatory challenges, and lucrative nature of coproduct development. Sustainable biofuel production could also have far-reaching societal impacts, especially in the context of our rapidly changing global climate. This perspective suggests that the path to profitable biofuel production is attainable and encourages consideration of similar approaches by the biotechnology industry.

**Graphical Abstract:**

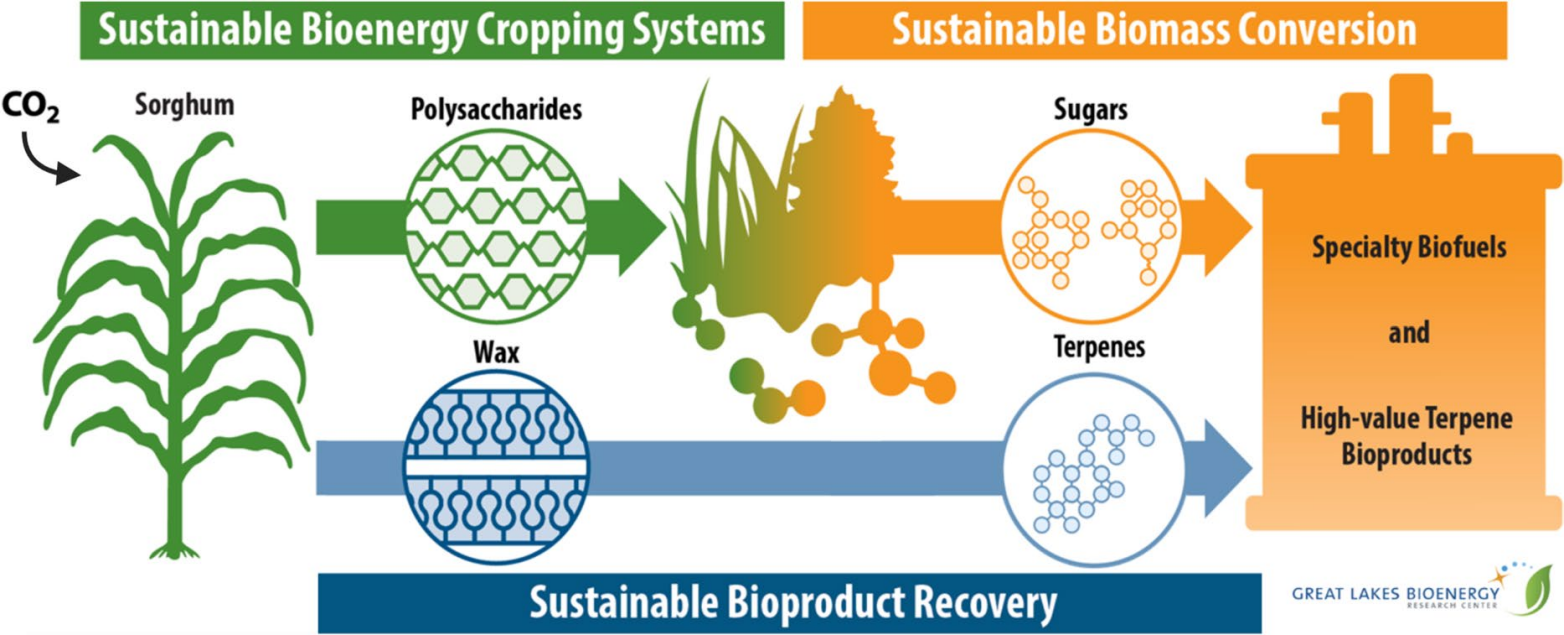

## Purpose and background

This perspective aims to provide (i) a rationale for biotechnological production of terpenoids as examples for high-value bio-(co)-products in engineered bioenergy crops, (ii) an emphasis on specific advantages *Sorghum bicolor* can offer as a biotechnological chassis organism and (iii) an evaluation of the opportunities, roadmap for commercialization, and framework of the potential business and societal impacts of these advances. We highlight technological and regulatory bottlenecks as well as comment on strategies and developmental research to overcome these obstacles.

For decades, researchers have explored different ways in which plants can be utilized to provide fuel and energy for an increasingly energy-demanding world. While plant-based biofuel production can decrease society’s reliance on fossil fuels, there is still ample opportunity to develop more cost-effective, sustainable alternatives to these traditional energy sources (Cherwoo et al., 2023). Similarly, innovation in providing sustainable small-molecule natural products is needed. Small molecule natural products are ubiquitous in industrial and consumer goods such as resins, agrichemicals, flavors, fragrances, pigments, cosmetics, nutraceuticals, and pharmaceuticals. These compounds are sourced from a natural host, biomanufactured, or synthesized in cell-free systems. The supply chains for procuring these compounds present many inherent challenges that prevent sustainable practices. These include the degradation of soil health, environmental fluctuations affecting agricultural productivity and global supply chains, reliance on fossil fuels for transportation and cultivation, the use of petrol-based chemical synthesis, the ecological contamination of synthetic derivatives, and a lack of purity of the intended product. These issues both lower accessibility and increase the required material input, limiting product sustainability.

To address these problems and expand access to natural products, sourcing small-molecule natural products through synthetic biology as bioalternatives has been successfully employed for various goods. Companies such as Manus Bio have implemented approaches at the cell level to extract the flavor compound nootkatone with less than 10 kg (kg) of input when typically, 400,000 kg of grapefruit is needed (Manus Bio BioAlternatives, 2024). Similarly, the companies Isobionics and BASF produce santalol, a sandalwood oil substitute, via fermentation with engineered microorganisms (Grzonka 2020). These synthetic biology approaches can also be extended to the pharmaceutical industry. Cell cultures of transgenic yew (*Taxus chinensis*) cells have been found to increase the production of the chemotherapy drug Taxol® by 1.7-fold (Zhang et al. 2011). At the whole-plant level, genetic transformation approaches have enabled the customizable production of beta-amyrin derivatives, which could confer distinct pharmaceutical properties (Reed et al. 2017). Moreover, genetic modification of wormwood (*Artemisia annua*) has increased the production of the antimalarial therapeutic artemisinin by up to 3.4-fold (Shi et al. 2017).

The use of synthetic biology in plants is a rapidly growing field that promises to provide high-value compounds while simultaneously enabling the fermentation of the remaining plant biomass into biofuels by existing infrastructure; however, commercial viability for these projects relies on robust technoeconomic analyses. Stable production of the vaccine adjuvant squalene in the bioenergy crop poplar was achieved; unfortunately, it was not price-competitive with commercial squalene unsustainably derived from shark liver (Bibik et al. 2024). Continued research, development, and adoption of these innovative approaches can fortify the supply of numerous compounds owing to the advantages that biomanufacturing and biochemical synthesis provide to the economic, environmental, and temporal sustainability of sourcing small-molecule natural products.

The examples highlighted above belong to the class of compounds named terpenoids. Terpenoids are specialized metabolites involved in the interaction of plants with their environment and are often associated with increased plant resilience. From an industrial perspective, terpenoids are attractive products because of their diverse range of pharmacological and nutraceutical properties and their applications in agriculture, cosmetics, flavors, and fragrances. Terpenoids are expensive and sometimes cannot be chemically synthesized or sustainably extracted from a native source (Leavell et al. 2016), making engineered plant hosts valuable for production.

To emphasize the importance of translational science and bring attention to an emerging biotechnology opportunity, we detailed the framework for the creation of a high terpenoid-producing sorghum variety to improve the economic viability of biofuels in the 2024 U.S. Department of Energy EnergyTech University Prize competition (EnergyTech University Prize, 2024). With support from the Great Lakes Bioenergy Research Center (GLBRC), we outlined a novel scientific project, production pipeline design, and the economic feasibility of this technology. In this review, we present a vastly expanded version of this work*.* We examine why sorghum is an intriguing choice for further study, highlight potential challenges and solutions to developing and cultivating hyperterpenoid-producing sorghum lines, and discuss the economic and societal impacts the success of this work could spark. By elaborating on this specific case study, we hope to emphasize the intrinsic value of considering future applications and implications of scientific works and encourage the broader adoption of this type of thinking*.*

## Why sorghum?

Sorghum has recently gained popularity as a bioenergy crop because of its resiliency in arid, nutrient-deficient environments (Hossain et al. 2022). This ancient grain was domesticated in Sudan approximately 4000–6000 years ago (Winchell et al. 2017), and as of 2023, the United States has emerged as the top sorghum-producing country in the world (USDA Production—Sorghum, 2024). Farmers typically choose to grow sorghum on perimeter lands unsuitable for other crops because of its ability to thrive in soils that would otherwise not be productive without significant agricultural input (Ngidi et al. 2024). These traits become especially relevant when large populations are fed in areas prone to extreme climactic conditions or with poor soil quality. The resilience of sorghum is in part due to its root system, which can branch widely and reach depths of more than 2 m, allowing water and nutrient scavenging at levels that are unattainable for many other crops (Lamb et al. 2022). Plants and their associated root systems are also the primary source of organic carbon found in soil, and this additional organic material deposition could offset greenhouse gas emissions and improve soil health (Dignac et al. 2017). We propose the use of the bioenergy sorghum cultivar R.07020 because of its ability to form particularly extensive root systems and therefore serve as a bioenergy source and a soil carbon sink (Ngidi et al. 2024). Bioenergy sorghum is a specific variety of sorghum bred for its long vegetative phase and high accumulation of above-ground plant tissue, primarily in the form of lignocellulose, the most abundant natural feedstock on Earth (Naraian & Gautam 2018). A recent analysis of sorghum’s potential for bioenergy production, with a focus on chemical composition and energy content, reported superior levels of non-structural carbohydrates over grass, or fiber crops (Ameen et al. 2024). Specifically for hybrid biomass sorghum, at a dry mass yield of about 35 Mgha^−1^, average contents of the main (structural) polysaccharides were found at 36–38% cellulose and 25–29% hemicellulose, next to 8–9% of lignin. These ratios are relevant for the saccharification following fermentation to produce second-generation ethanol, with lignin content lower than that of sugar cane bagasse at a variable 11–25% (Almeida et al. 2019). As an annual crop, sorghum also provides farmers with flexibility and allows for rotation, which contrasts with other bioenergy crops such as the perennials switchgrass and poplar.

Compared with other plant species, sorghum also produces large quantities of wax in its cuticle, which substantially reduces water loss, provides a highly hydrophobic environment and contributes to its resilience (Xiao et al. 2020). The sorghum cuticle naturally accumulates significant amounts of C30 triterpenoids, including the alcohols β-amyrin, α-amyrin, fernenol, fernenone, isoarborinol, isoarbornone, and semiarenol (Busta et al. 2021). This phenomenon is specific to sorghum and is not found in related bioenergy species such as maize. Targeting the production of hydrophobic high-value terpenoids to the epidermis of sorghum would provide an ideal environment for the sequestration of bioproducts in the cuticle and enable an efficient downstream method for purification*.* The wax layer is removed before downstream processing of the lignocellulosic biomass, i.e., separation of the cellulose, deconstruction to sugar, and fermentation to ethanol or higher fuel types. This physiological feature of sorghum permits overcoming a major technological and economic bottleneck of biotechnological production: simplifying the separation of the primary product from the chassis biomass. In contrast to traditional solvent-based extraction, bioproducts are harvested from wax via supercritical CO_2_ extraction to yield high-value food-grade compounds, sorghum wax, and other plant materials. This process typically recovers and recycles a large proportion of the CO_2_ used in extraction, minimizing its carbon footprint and eliminating solvent in the residual biomass (Montesantos & Maschietti 2020). A leading pioneer of the global sector for natural product extraction with supercritical CO_2_, Phasex (Phasex Corporation, North Andover, MA, USA) has operated for nearly five decades with a focus on terpenoid extraction and purification and has considered pilot scale wax and terpenoid extraction from sorghum as feasible. Specifically for squalene, extensive studies have established and optimized supercritical CO_2_ extraction conditions (pressure, temperature) for various plant sources, including Amaranth. Comparison with conventional solvent-based methods (e.g., Soxhlet, hexanes, petrol ether) found supercritical CO_2_ extraction yield of squalene and broad applications of the byproducts in the food industry more cost-effective and environmentally friendly (Popa et al. 2015). Experimental parameters for diterpenoid extraction using supercritical CO_2_ were established in general and recently reported (Uwineza & Waśkiewicz 2020), with abienol exclusively detected in these extracts across different procedures (Duquesnoy et al. 2010). Modern varieties of sorghum planted in agriculture are elite hybrids selected and bred for superior traits. Consulting with sorghum breeding programs such as those at Texas A&M University, Kansas State University, Purdue University, or the USDA Agricultural Research Service (ARS) is highly recommended prior to establishing new traits in sorghum cultivars to ensure compatibility for trait introduction into the seed parent lineages for generation of the hybrids for commercial application. Overall, sorghum is a resilient crop that can greatly facilitate the sustainable production, sequestration, and extraction of desirable compounds, while the remaining biomass can be converted into biofuels.

## Terpenoid bioproducts as targets

An inspiring study suggested that the coproduction of bulk industrially relevant feedstocks and specialty bioproducts can positively impact the economic viability of biofuels (Yang et al. 2020). In this analysis, we focus on two pilot bioproducts: squalene and *trans*-abienol. The biosynthetic pathways for both products are short and well understood. Squalene is a vaccine adjuvant and cosmetic ingredient with the current cheapest source extracted from shark liver oil. *Trans*-abienol is a precursor for the semisynthesis of ambroxides, which are valuable amber odorants in the fragrance industry (Johnson et al. 2019). Production of squalene has been reported in stably engineered poplar lines, including a technoeconomic evaluation and estimated threshold for commercial competitiveness (Bibik et al. 2024). The route to heterologous accumulation of *trans*-abienol was earlier demonstrated in the transient *Nicotiana benthamiana* system, and the process was reproduced here (Johnson et al. 2019). Optimizing squalene and *trans*-abienol production in the sorghum epidermis and sequestration in the cuticle would enable efficient and stable generation of these high-value, small-molecule natural products.

## Results

### Identification of tissue-specific promoters/regulatory elements

The tissue-specific expression of terpenoid biosynthesis genes is necessary for the targeted production of high-value biomolecules. Accumulation in the cuticle can circumvent the potential adverse side effects of perturbation of metabolic homeostasis in unrelated tissues and developmental stages. For efficient production, the identification and characterization of promising promoters, and subsequently putative *cis*-regulatory elements (pCREs) will deliver the tools to achieve high terpenoid yields within the epidermis, or any other tissue or cell-type subjected to targeted metabolic precision engineering. In model plants, a palette of molecular methods developed over decades has enabled the discovery of biotechnologically relevant sequences carrying regulatory elements. An illustrative example is the discovery and characterization of W-boxes, using transient expression in a parsley-elicitor system nearly 30 years ago. The short CREs were found to trigger rapid response to fungal elicitation, coordinated by sequence-specific DNA-binding proteins (WRKY transcription factors) (Rushton et al. 1996). W-boxes and WRKYs are now recognized as key players mediating multifaceted aspects of plant resilience, including regulation of commercially relevant terpenoids and specialized metabolism. Their actions have also been demonstrated in the sorghum pericarp (Schumaker et al. 2024). Sorghum transformation technology has received significant attention yet largely still relies on decades-old, unregulated, and inherently problematic promoters, such as rice Actin1, cauliflower mosaic virus 35S and maize UBI1 (Silva et al. 2022), throttling biotechnological progress. New methods in adoption for sorghum include DNA Affinity Purification Sequencing (DAP-seq), which can generate genome-wide binding maps for in vitro expressed transcription factors (TFs), or in vivo Assays for Transposase-Accessible Chromatin using Sequencing (ATAC-seq), identifying open, accessible chromatin regions (Fontanet‐Manzaneque et al., 2024; Zhou et al. 2021).

Tissue and cell-type specific gene expression data allows for initial identification of promoter candidates for targeted expression in sorghum. Candidate genes were identified in sorghum gene expression data from RNA-seq libraries generated via laser capture microdissection (LCM) from the sweet sorghum variety Della (Fu et al. 2023). Genes expressed with high epidermal specificity compared to other stem tissues (bundle sheath, fiber tracheid, phloem, pith, and xylem) were identified through a Tau analysis of the Della LCM data (t > 0.9) and filtered for stem epidermal expression ≥ 20 TPM. Genes were then selected for expression of > 10 TPM in the middle of juvenile leaves and < 10 TPM in the top and bottom of juvenile roots in the sorghum variety BTx623 (Phytozome 13). Initial candidates include Sobic.003G069200, Sobic.006G229300, Sobic.007G132000, Sobic.002G122000, and Sobic.003G069200. Sequences upstream of the start codon (< 5000 bp) of the identified genes were enriched with binding sites for WRKY TFs (≥ 18 WRKY TFmatrixIDs; PlantPan 3.0) and TFs in the ethylene response factor (ERF) family (> 25 ERF TFmatrixIDs; PlantPan 3.0), except for Sobic.003G069200 (5 ERF TFmatrixIDs). ERFs are involved in the regulation of genes in response to biotic and abiotic stresses, which often occur at leaf surfaces, and ERFs have been found to be associated with the leaf epidermis in maize (Moose & Sisco 1996). Utilizing both wet and dry lab techniques, it is possible to achieve large-scale prioritization of candidate putative *cis*-regulatory elements and reduce the time and resources typically required for functional validation.

The last decade has seen a rise in the use of dry lab approaches such as artificial intelligence (AI) approaches in genomics and synthetic biology (Farooq et al. 2024; Harfouche et al. 2019; Helmy et al. 2020; Iram et al. 2024; Rai 2022; Xu et al. 2021). Here, we propose the use of a type of AI approach, machine learning (ML), to identify pCREs potentially responsible for targeted gene expression in sorghum. By utilizing ML, we can create a directed list of pCREs to test, reducing the number of potential *cis*-regulatory elements requiring experimental validation. The methodology relies on an approach that previously identified pCREs for cold-responsive genes in switchgrass via a Random Forest (RF) classification model that has been shown to be effective at identifying both pCREs and experimentally validated CREs (Ranaweera et al. 2022).

**Fig. 1:**
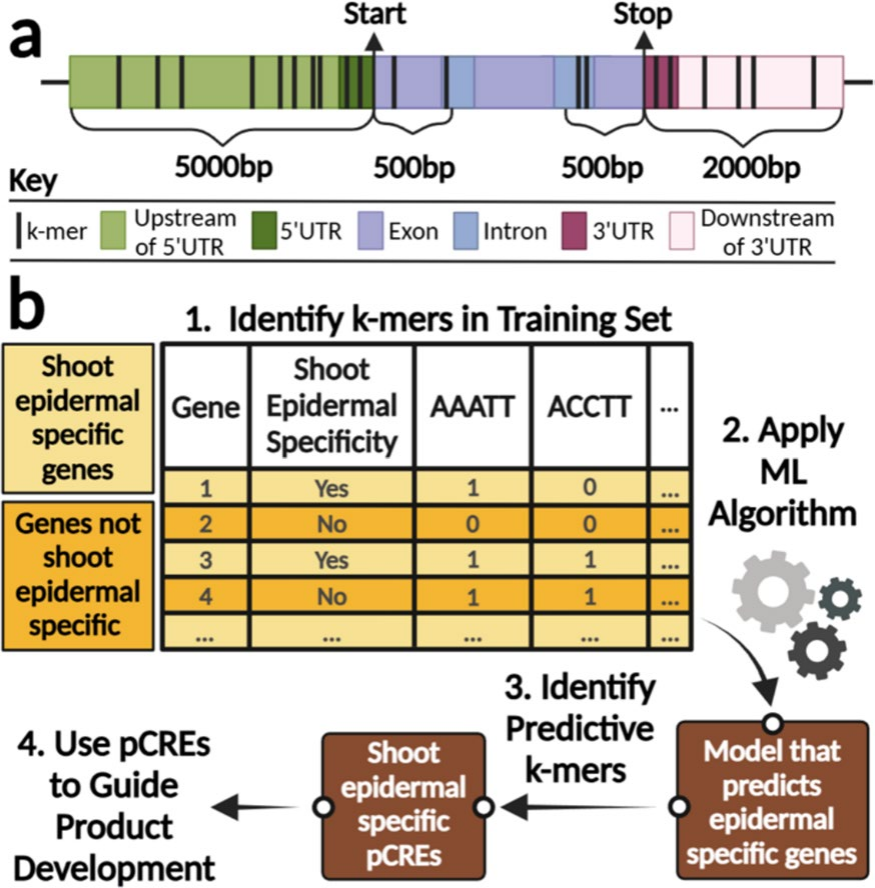
Machine Learning (ML) pipeline for identification of k-mers likely to be sorghum shoot epidermal specific pCREs. **a.** Regions for k-mer identification in relation to the start codon (Start) and stop codon (Stop) of each gene. **b.** The pipeline for identifying pCREs with the ML model. **b.1**. Identify k-mers that are between 5 and 9 bases long within the regions depicted in a. **b.2.** The ML algorithm is applied to the k-mer dataset to develop a model that predicts epidermal specific genes. b**.3.** The model can then be used to identify k-mers likely to be shoot epidermal specific pCREs. **b.4.** The pCREs can then be used to guide targeted production of high-value terpenoids

To identify key pCREs of genes that have high sorghum shoot epidermal specificity, we will first conduct a k-mer enrichment. Initially, potential pCREs (k-mers) will be identified from two regions (Fig. 1**.a.**), i) sequences 5 kb upstream and 500 bp downstream of the gene start codons, as well as ii) 2 kb downstream and 500 bp upstream of the stop codons, from the *Sorghum bicolor* v3.1 annotated genome (McCormick et al., 2018). Each k-mer is between 5 and 9 bases long, inclusively. When training the model, the two classification groups are genes that have high shoot epidermal specificity (positive class) and genes that do not (negative class; Fig. 1**.b.**). Specifically, the positive training set will consist of genes with high stem epidermal specificity (compared with other stem tissues: bundle sheath, fiber tracheid, phloem, pith, and xylem), expression in juvenile leaves, and low root expression (gene expression data, Phytozome).

An RF classification model will be built with the training data to identify genes with high epidermal specificity (Fig. 1**.b.**). The best model is determined based on the F1 score of 5 cross-validation folds. After this, the minimum number of k-mers that can produce a similar performing model to the best RF model will be identified. The identification of k-mers to include will be based on the Gini importance, which indicates the contribution of a k-mer in distinguishing between two classes. The efficacy of the identification of epidermal-specific genes with k-mers will be checked with the testing set after the model has been validated during training. Once k-mers are identified, additional analysis, as described by Azodi et al. (2020), will be conducted to identify which k-mers are a part of previously identified transcription factor-binding sites and regulatory elements in sorghum, after which the k-mers will be identified as pCREs (Azodi et al. 2020).

**Fig. 2:**
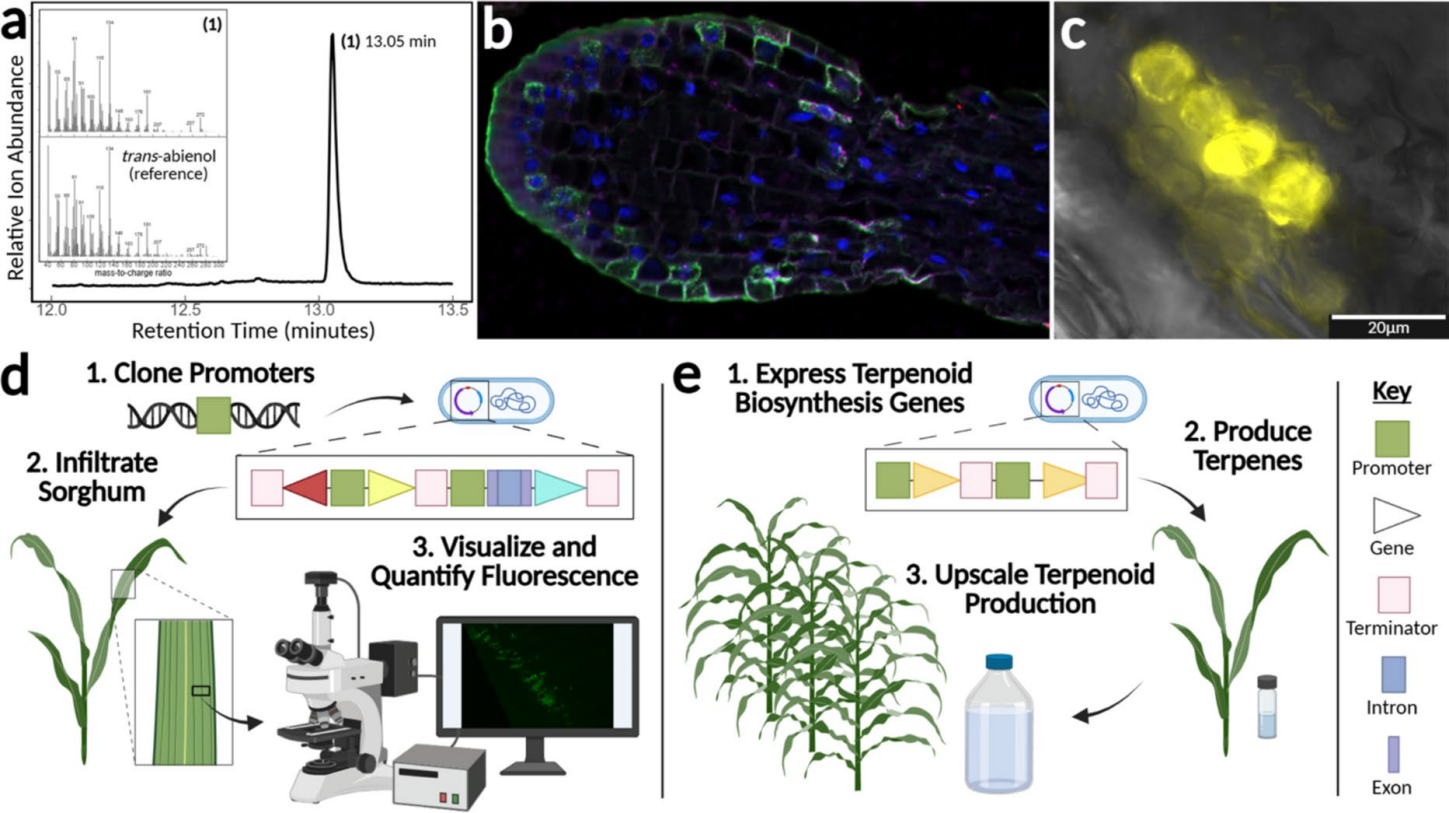
Steps for targeted production of *trans*-abienol in stably engineered sorghum. **a.** Engineered expression, and detection of the diterpene *trans*-abienol pathway (*Plectranthus barbatus* TPS2, *Origanum majorana* TPS3) in *Nicotiana benthamiana*, experimentally reproduced from Johnson et al., (2019). GC/MS Total ion chromatogram of hexane extract, (1) *trans*-abienol, insets MS spectra of (1) and reference spectrum of authentic standard. **b**. RNAscope ISH assay, *in-situ* hybridization of longitudinal section of sorghum root tip. Channels (i) green, positive control, potential glutathione S-transferase identified through tau-analysis (Sobic.001G280800.1, root hair, TPM mean 581), (ii) pink, O-methyl transferase 3 of the sorgoleone pathway, (Sobic.006G007900.1, root hair, TPM mean 280), (iii) blue, nuclei, DAPI stain. Negative control, *Bacillus subtilis* DapB. Image size, 40X. Multiplex v2, RNAscope® assay (Bio-Techne, MN, USA), University of Wisconsin, Department of Pathology and Laboratory Medicine. **c.** Transient expression of YFP driven by the *Zm*UBI promoter in young sorghum leaf tissue, experimentally reproduced from Sharma et al., and Miller et al. (Miller et al. 2023; Sharma et al. 2020). **d.** Testing promoters in sorghum via Agrobacterium-mediated transient expression. **d.1.** Clone promoters from sorghum genomic DNA and insert into a transient expression vector between super-TagRFP (red gene) and *Amil*GFP (yellow gene) and transform into Agrobacterium. **d.2.** Infiltrate sorghum with Agrobacterium containing the transient expression vector. **d.3.** Visualization and quantification of the fluorescence of *Amil*GFP and super-TagRFP normalized to *Am*Cyan (cyan gene) driven by the *Zm*UBI promoter. **e.** Development of stable terpenoid-producing sorghum lines. **e.1.** Clone the terpenoid biosynthesis genes and insert into the plant expression vector under shoot epidermal-specific promoters validated in a. and transform into Agrobacterium to express terpenoid biosynthesis genes. **e.2.** Test tissue-specific production of terpenoids by transient expression via infiltration in sorghum. **e.3.** Stably transform tested terpenoid biosynthesis genes into sorghum and grow transgenic sorghum in the field to increase terpenoid production

The test set will have a mix of genes that may or may not have information about high shoot epidermal specificity and may include genes whose promoters have been experimentally validated for sorghum shoot epidermal specificity as described below (Fig. 2**.d**.). The promoter regions (up to 5 kb upstream of the start codon) of these candidate genes will be cloned and tested for shoot epidermal specificity via transient expression in sorghum. This will enable the identification of smaller regions of the promoter that are pCREs, with the goal of identifying a minimal functional part sufficient to control engineered transcriptional units for the rational design of sorghum lines.

### Sorghum transformation and validation of pCREs

Natural transgenes with expressed Agrobacterium transfer DNA (T-DNA) sequences have existed since the cultivation of one of our oldest crops, sweet potato (Kyndt et al. 2015). In contrast, “unnatural” or intentionally designed and engineered transgenic plants did not emerge until the discovery of Agrobacterium-mediated transfer and expression of heterologous genes in tobacco four decades ago (Herrera-Estrella et al. 1983). As of June 2024, the International Service for the Acquisition of Agri-biotech Applications (ISAAA) listed 32 approved gene-modified crop species and a total of 505 events (i.e., individual lines) globally, including the biomass species maize, poplar, and sugarcane (GM Crops List, 2024). While sorghum is not yet on the list, significant technological advances are now enabling U.S. transformation facilities to offer sorghum transformation services, including the Danforth Plant Science Center and the Universities of Missouri, Rhode Island, Wisconsin-Madison, and Texas Tech. Breakthroughs include increased tissue culture regeneration capacity and consequently transformation efficiency, increased genome editing frequency and even transformation of leaf tissue, circumventing the notoriously time-consuming approach of relying on immature embryo tissue culture (Parrott et al. 2024). However, sorghum transformation remains far from a routine technology. Here, details related to the commercial viability of sorghum transformation with a focus on metabolic engineering and the coproduction of high-value terpenoid bioproducts are provided. Specifically, the requirements for transformed events are rapidly becoming more complex, including necessities for multigene delivery or multiplexed simultaneous gene editing. It has also become increasingly clear that unregulated constitutive promoters are far less than ideal for driving desired traits because of potential problems such as regeneration of plantlets, unspecific phenotypes, and an overall lack of control of transgene expression (Bibik et al. 2024).

The earliest stable transformation of sorghum occurred in the 1990s and involved the electroporation of protoplasts or bombardment with DNA-coated microprojectiles (Battraw & Hall 1991; Hagio et al. 1991). Agrobacterium-mediated transformation of immature embryos was demonstrated a decade later with an herbicide selectable marker (Zhao et al. 2000) and remains the dominant technology. The Wisconsin Crop Innovation Center (WCIC), a public crop biotechnology service and research center, provides agrobacterium-mediated sorghum transformation as a fee-for-service. According to WCIC, a single transformation and onboarding of our proposed sorghum bioenergy cultivar R.07020 will cost $20,000. After this cultivar is established, the fee for the external public (University and USDA) for transformation is $7,168 as of June 2024, but additional costs will accrue for vector cloning, vector transformation into Agrobacterium, event genotyping and bulking up of seeds to yield an approximate total of $8,518. These subsequent transformation prices are for delivery of T1 seeds from up to 6 independent events/constructs with an average turnaround time of 8–12 months (Wisconsin Crop Innovation Center Homepage 2024). As of April 2025, the two sorghum cultivars available for transformation by WCIC are both inbred grain types, RTx430, and Tx623. So, adoption of the proposed bioenergy cultivar will also represent a significant technological advance for the scientific community, as other groups will have a lower barrier of entry for transformation.

Transient transformation, or the temporary delivery of gene constructs without stable integration into the host genome, is used in plant synthetic biology to accelerate testing various aspects of transformation including functional parts, delivery and target chassis. While of intense interest, this technology is currently in its infancy for sorghum and other bioenergy plants. Yet, the adoption of engineering traits, including terpenoid biochemistry, can benefit from successes in model plant species. Here, critical steps towards this goal are illustrated. Two teams independently established methods for Agrobacterium-mediated transient expression in sorghum via infiltration (Miller et al. 2023; Sharma et al. 2020). The transient transformation of sorghum has proven sufficiently robust, setting the stage for systematic tweaking of the experimental parameters (Fig. 2**.c**). In addition, the parts, vector and experimental procedure for transient heterologous expression and detection of *trans*-abienol were reproduced, serving as guide for transfer from the model species *Nicotiana benthamiana* (Johnson et al. 2019) to sorghum (Fig. 2**.a**). The procedure for cell-type specific detection of sorghum transcripts in epidermal cells can provide indirect evidence supporting selection of the related promoter region (Fig. 2**.b**; note, root epidermal tissue is shown). Next, optimizing transient expression allows the testing of regulatory elements and subsequent terpenoid production in sorghum without the arduous and time-consuming task of stable transformation. A vector designed for testing promoters in sorghum will include two fluorescent proteins codon optimized for sorghum, super-TagRFP (Mo et al. 2020) and *Amil*GFP (Alieva et al. 2008), on either side of the promoter insertion site for assessment of bidirectionality or leakiness of the promoter (Fig. 2**.d.**). Once terpenoid production is established transiently, sorghum can ultimately be stably transformed to produce high-value terpenoids (Fig. 2**.e.**).

Experimentally, a third codon-optimized fluorescent protein, *Am*Cyan, is driven by the *Zea mays* ubiquitin (*Zm*UBI) promoter, which is constitutively active in sorghum (Able et al. 2001). This addition will enable normalization of *Amil*GFP and super-TagRFP to *Am*Cyan for quantification and it will ensure visualization of all successfully transformed cells. Moreover, excitation of *Am*Cyan with a flashlight of the appropriate wavelength and visualization with a filter to remove autofluorescence will allow for expedited screening of transient expression before promoter testing. An intron upstream of *Am*Cyan reduces the potential for expression of the fluorescent protein within Agrobacterium.

Indirect support for the activity of a candidate gene promoter can be provided by tissue- and cell-specific localization of the respective target mRNA through in situ hybridization (ISH) (Fig. 2**.b**). While routinely applied in pathological medicine, service facilities such as the Translational Research Initiatives in Pathology (TRIP, University of Wisconsin–Madison) can use fluorescent ISH with up to four colors for multiplexing, compatible with plant tissues (University of Wisconsin–Madison Department of Pathology and Laboratory Medicine 2024). A standard single ISH is currently charged at $113 per slide, with each additional probe at $39.50 per slide.

### Market sizes and technoeconomic perspectives

A total of 6.4 million acres of sorghum were planted in the United States in 2024, and annual sorghum acreage has increased each year since the pandemic in 2020 (USDA Production—Sorghum). The Food and Agriculture Organization of the United Nations predicts an increase in sorghum production globally through 2027 (Stamenković et al. 2020). These trends suggest that sorghum is increasing in prevalence and importance, likely because particular sorghum varieties have significant agricultural and technological potential. One such area of potential is the use of bioenergy sorghum for biofuel production, though it requires further development to enable commercial scale production. To aid with the scaling up process, the biofuel industry can leverage government subsidies to compete with fossil fuels; however, even with existing infrastructure, biofuels maintain a niche role in the American energy economy. In 2023, approximately 9% of the energy output of the United States came from renewable energy, and of that, biofuels constituted 32% of renewable energy (U.S. Department of Energy 2024; U.S. Energy Information Administration 2024). The technology discussed in this paper aims to increase sorghum biofuels within the biofuel ecosystem by coupling agricultural energy production with the synthesis of valuable flavor and fragrance chemicals. This model could help sustain the growth and viability of the biofuel industry by providing a parallel source of revenue and therefore an incentive to grow sorghum for biofuels.

**Fig. 3:**
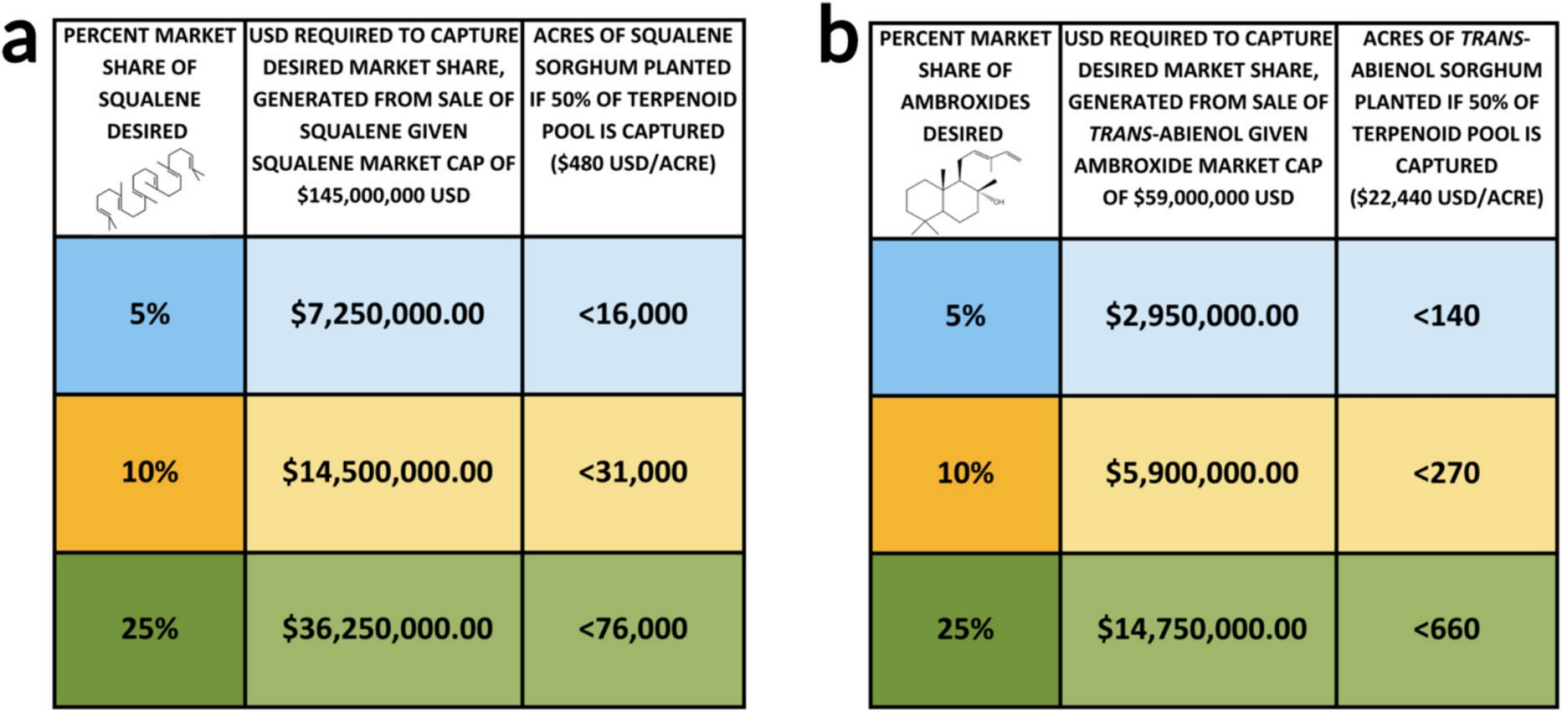
Overview of the acres of terpenoid-producing sorghum needed to achieve a specific market share of the squalene and ambroxide market given the proportion of the available terpenoid pool captured. **a.** Projections for bulk chemical squalene given 50% capture of the available terpenoid pool. **b.** Projections for the bulk chemical trans-abienol given 50% capture of the available terpenoid pool

The global flavors and fragrance industry (including end products) was valued at $1.4 trillion in 2019 and was projected to grow to $1.8 trillion by the end of 2024 with a compound annual growth rate (CAGR) of 4.0% (BCC Publishing 2020). The ability to grow such a large market is attributed to the diversity and robustness of the flavor and fragrance industry. High-value terpenoids, one of the dominant classifications of flavors and fragrances, can be viewed as either bulk or specialty chemicals. Bulk chemicals demand a greater volume to maintain the industries and distributors reliant on them and have the potential to drive up the cultivation of sorghum for biofuels. Specialty chemicals are difficult or expensive to synthesize, often resulting in cost limitations for expansion of the market. The plants producing these specialty chemicals will be cultivated on less land to avoid diluting market prices but will still be an asset for the growth and diversification of the production platform. Two key markets, squalene and *trans*-abienol, present opportunities for sorghum to emerge as a major producer of these small molecules. Squalene, a bulk chemical, has a market cap of $145 million with a CAGR of 10.5% through 2032 (Polaris Market Research 2024). The specialty chemical ambroxide, of which *trans*-abienol is a precursor, had a relatively smaller market cap of $59 million USD in 2023 and a projected CAGR of 1.7% through 2031 (MarketsGlob 2024). There are countless other terpenoid markets to which this technology could be applied, but focusing on those where the products are supply-limited is a promising initial step. Considering both the market growth and anticipated yields of these two example compounds, there is potential to capture a sizeable market share even while cultivating sorghum on limited land (Fig. 3).

The biomass yield of bioenergy sorghum can range from 10–48 Mg/ha (Lamb et al. 2022), and it is estimated that approximately 100–200 kg of cuticle wax per hectare of sorghum can be recovered and used for small molecule extraction (Chemelewski et al. 2023). A significant carbon allocation in the largely non-photosynthetic epidermal cells has been established throughout the developing hydrophobic cuticle. With a load of 8.0 ± 0.8 μg/cm^2^ leaf surface, terpenoids were found to amount to nearly 40% of the total carbon deposit in the cuticle of adult stage sorghum (Busta et al. 2021), reached at 25–49 days after planting. On the basis of isoprenoid pool projections and publicly available prices for squalene and *trans*-abienol ($40/kg for squalene and $1.87/gram for ambroxides), estimates indicate that even the cheaper squalene product would achieve cost parity of a biorefinery as modeled by Yang et al. (2020) if squalene accumulated at 50% of the same yield as native triterpenoids (0.086% of dry weight harvested biomass) (Barrero et al. 1993; Bibik et al. 2024; Perfumer’s World 2024; Yang et al. 2020). This would equate to approximately 12 kg/acre of product and $480/acre for the value of squalene alone (Bibik et al. 2024). In the case of *trans*-abienol, accumulation at 50% of the same yield as native triterpenoids would equate to approximately $22,440/acre for the value of the processed ambroxide product, greatly exceeding necessary profit margins for even target minimum ethanol selling prices (Barrero et al. 1993; Perfumer’s World 2024; Yang et al. 2020). These projections complement the current value of bioenergy sorghum biomass alone and could be the key to unlocking sorghum biofuels as an economically viable sector of the biofuel industry.

Scaling of engineered sorghum lines involves starting with established markets, like squalene and *trans*-abienol, and then executing research and development in parallel to break into new markets. By diversifying into both bulk and specialty terpenoid portfolios, one can avoid market saturation and a consequential drop in the price of more common terpenoids, such as squalene, while simultaneously expanding markets for terpenoids that are difficult to synthesize, such as *trans*-abienol. This will also deescalate risk with newer terpenoids while leaving room for expansion with more ready-to-go compounds. The envisioned long-term goal will be to maintain smaller stakes in many markets, resulting in a large portfolio that is greater than the sum of its parts. When considering expanding both production and refining operations, geographic barriers are present. Current models of biofuel profitability assume the use of marginal land which is not profitable for food crops for a variety of reasons. These include poor soil quality and depth, a lack of micro- and macronutrients, and/or arid conditions (Hossain et al. 2022). Unsuitable for food production, these regions are ideal for the cultivation of bioenergy crops. Testing multiple models for country-wide scaling, biofuel production was predicted at $4/gallon. The sustainable scenario reached the threshold despite limiting the bioenergy crop area to the Eastern Continental United States and regions with rainfall (Cai et al. 2011; Uludere Aragon et al. 2022). Another challenge to be addressed is the narrow geographic distribution of existing biorefineries, which are co-located with existing bioethanol production facilities. Prior work demonstrated that reducing the distance between biomass and refining infrastructure directly improves the economic viability of the resulting biofuels (Ng & Maravelias 2017). Hence, a network of biorefineries that geographically mirror areas of marginal land allocated to bioenergy crops will provide leverage to build an efficient supply chain. Together, these practical and economic considerations point towards critical needs and opportunities for the development of bioenergy crops with co-production of high-value bioproducts.

### Risk assessment and regulation

Initial field trials will assess environmental safety and performance of experimental transgenic lines in the inbred sorghum variety established for routine transformation. Future agricultural applications for commercialization of the technology will need to assess in depth regulation regarding release of transgenic material, consider stakeholder input and transfer the trait into a commercially relevant genetic background of elite hybrid varieties.

From the perspective of regulation, controlling and mitigating the risks associated with introducing novel transgenic crops is necessary to minimize dispersal of a genetically modified organism (GMO) or its pollen. The risks associated with the use of genetic engineering of bioenergy crops are in part mitigated by the suggested system: the sorghum biomass is not used for food or animal feed, minimizing exposure to humans; harvesting before the development of reproductive organs and the setting of seeds, or pollen production prevents dispersal of recombinant genetic material. The proposed experimental R.07020 cultivar of bioenergy sorghum is well suited for this practice because it is photoperiod sensitive and flowers late in the season, or not at all, due to early frost in the northern Unites States, thus allowing for a longer vegetative growing period to maximize biomass accumulation and preventing the setting of seeds in certain growing zones (Casto et al. 2018). Seed stocks are generated from plants in greenhouses in a controlled environment, and the regulatory authority is the United States Department of Agriculture-Animal and Plant Health Inspection Service (USDA-APHIS). Central for field trials of transgenic crops is the evaluation of risk that engineered traits can pose upon exposure of native species to transgenic material, including an altered fitness in, e.g., noxious agricultural weeds such as *S. halepense* (“Johnsongrass”), or shattercane (*S. bicolor* ssp. *drummondii*) which readily introgress with *S. bicolor*. Existing strategies preventing outcrossing include engineered male sterility (i.e., no transgenic pollen), and physical separation or cultivation in climate zones preventing either pollen formation (photoperiod sensitive varieties) or where no compatible native species are present. Field trials have already been approved and conducted for transgenic sorghum metabolically engineered for 4-hydroxybenzoic acid production under the USDA-APHIS Biotechnology Regulatory Services (BRS) (Lin et al. 2022). In general, research institutions handling scientific evaluation of transgenic plants and material generated in any of the fee-for-service facilities, or at other academic institutions, are familiar with the APHIS-BRS mission. APHIS provides the regulatory oversight of certain genetically modified organisms outside of the jurisdiction by U.S. Food and Drug Administration and the U.S. Environmental Protection Agency. Specifically, an APHIS permit is already required for interstate movement and importation of regulated genetically engineered organisms (as defined in Code of Federal Regulations, 7 CFR § 340.1). Key determining language in the context of the regulatory review for the release of genetically engineered organisms is the assessment whether there is a plausible route by which any sexually compatible, and for sorghum specifically, weedy relative, can acquire the engineered trait and, would pose an increased (…) risk. For additional information the reader is referred to the informative ‘Am I Regulated (AIR) Process Guide for Submission of AIR Inquiries’ on the APHIS website (https://www.aphis.usda.gov/biotechnology/am-i-regulated).

From the perspective of the stakeholders, e.g., the US sorghum growers and producers association (National Sorghum Producers, NSP), a possible contamination of non-GMO sorghum for international export (U.S., 5.4 million tons, 2024) through pollen drift from engineered sorghum varieties remains a concern. Eliminating possible gene-flow from engineered varieties into grain sorghum in agricultural settings, however, is addressed through multiple barriers including physical (cultivation on marginal, non-agricultural land), biological (male sterile lines to prevent pollen flow, and lines that delay or prevent flowering under a temperate photoperiod (i.e., photoperiod sensitive)) and general compliance with USDA-APHIS regulation for transgenic material.

From the perspective of the breeder, the development of elite hybrid bioenergy lines carrying the engineered traits and supplying seeds for agricultural applications and ultimately commercialization, is the goal. Instead of cumbersome introgression of a transgenic inbred into elite background, followed by backcrossing, an attractive strategy is integration of the transgenic trait into the (female) seed parent variety followed by crossing with the commercially relevant pollen donor(s) to create hybrid biomass lines with agricultural traits determined by the male line(s). In sorghum agriculture, conventional breeding and (hybrid) seed production is the only current pipeline. Integration and processing of transgenic traits into elite background remains strictly separate and is limited in capacity by few facilities with infrastructure to accommodate engineered varieties (ARS/Kansas/Purdue/Texas A&M). Building opportunities for this industry will require investment in dedicated facilities for molecular work, i.e., vertically integrated transformation and scaling of seed production and distribution, compliant with transgenic regulation.

The proposed terpenoid products, squalene and *trans*-abienol do not require a specific premarket approval by the Food and Drug Administration (FDA), as they are already utilized as cosmetic ingredients (21 USC Ch. 9: FEDERAL FOOD, DRUG, AND COSMETIC ACT, 1938), upheld by supportive precedents in modern jurisdiction (*Franz v. Beiersdorf, Inc. *et al*.*, 2015) and due to substantial equivalence policy regarding products generally recognized as safe (GRAS). As a final consideration, transportation of the terpenoid products, either by rail or road, does not pose a significant risk, especially if the leveraging of existing infrastructure is optimized, and lessons are learned from biofuel ethanol, given the latter’s challenges in bulk transportation (National Transportation Safety Board 2018).

### Societal impacts

Assuming success and an abundance of sorghum-derived terpenoids produced, one critical societal impact that this project envisions is a reduction in carbon emissions from fragrance production, among other industries. Currently, more than 486,000 tons of fragrance are derived from petrochemistry annually, accounting for 76% of the fragrances employed by the fragrance sector (Elterlein et al. 2024). This project offers a promising alternative to petrochemistry for synthesizing high-quality, eco-friendly fragrances. Implementing this system would allow the industry to avoid the carbon emissions of petrochemistry and lessen their detrimental contributions to climate change. In addition, as of 2024, any transgenic plant residue can be effectively devitalized by composting instead of the energy-demanding autoclaving process (Standard Operating Procedure (SOP) Template for APHIS BRS Plant Permits 2024).

Generally, on an energy-equivalent basis, biofuels still have higher production and retail costs than their fossil fuel counterparts because of processing and scaling requirements (Alternative Fuel Price Report April 2024; Economics of Biofuels, 2024). This project could improve socioeconomic equity by making the price of biofuels competitive and lessening the burden of climate change on disadvantaged communities by increasing access to sustainable energy. With the proposed coproduction of high-value compounds in the sorghum cuticular wax, post-terpenoid extraction bimass can be recaptured, reducing resource intensity of biorefineries, ultimately decreasing fuel costs. Farmers growing sorghum will also benefit from this coproduction model through government subsidies in the form of value-added producer grants. These grants provide farmers with a reimbursement of 50% of the cost of establishing a biomass feedstock crop, as well as annual payment for up to five years for herbaceous feedstocks (U.S. Department of Energy).

The proposed project will also have positive impacts on soil health. Under proper soil management practices, sorghum cultivation generally leads to increases in soil organic carbon, a primary indicator of soil health (Meki et al. 2013; Presley et al. 2012; Sainju et al. 2015; Stockmann et al. 2013).

**Fig. 4:**
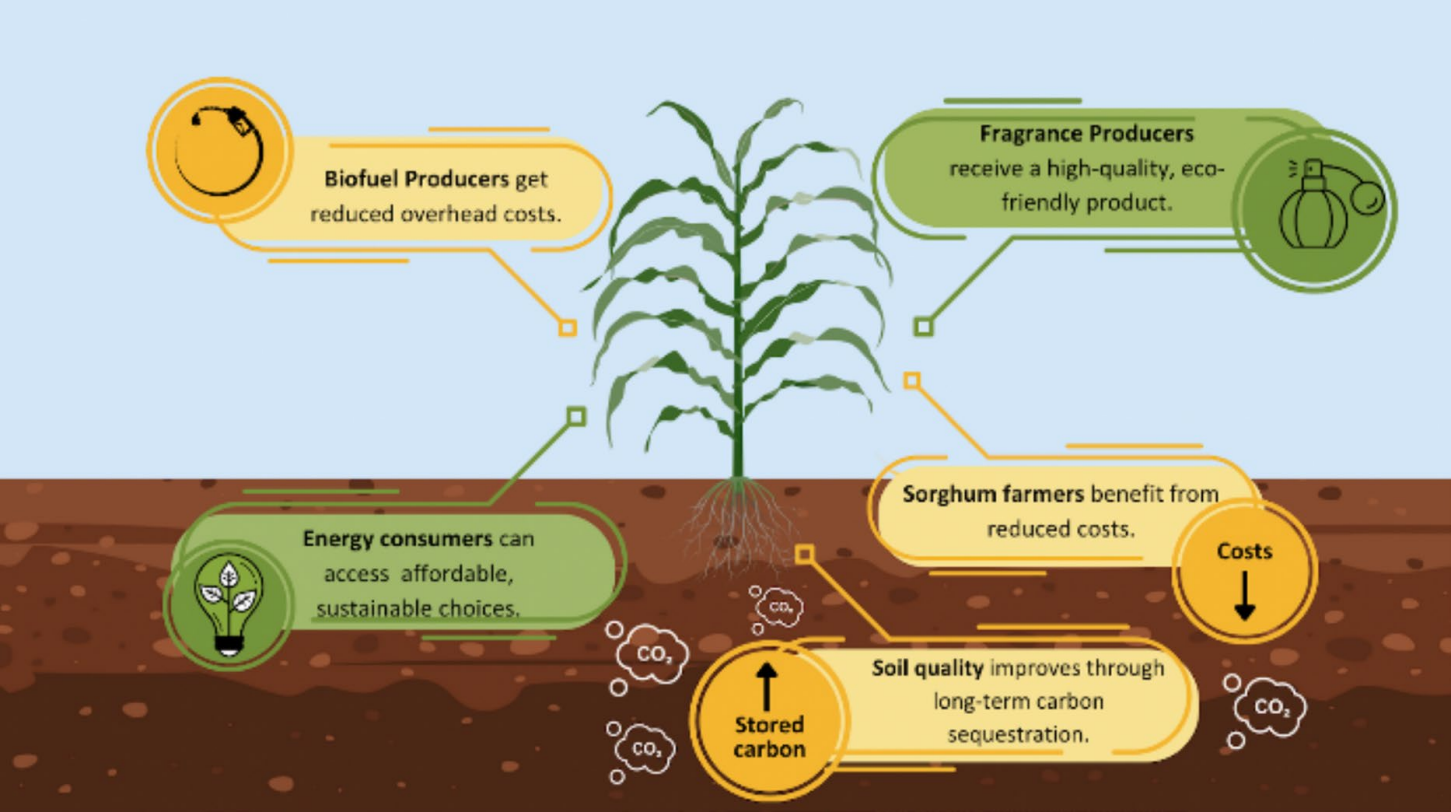
In this system, the depleted wax, extracted for the desired high-value compounds, is changed from a traditional waste product into a commodity, whereas processed sorghum residue is upgraded into a feedstock. Extracted sorghum biomass can enter the biorefinery conversion supporting efficiency and cost-competitiveness of the biofuel. The value-added producer grants that sorghum farmers will benefit from will decrease their costs, and the quality of the soil on which sorghum grows will improve through carbon sequestration and increases in soil organic carbon levels

The proposed sorghum-based platform for terpenoid production will harvest aerial biomass and leave a.large quantity of organic matter in the soil, thus increasing soil organic carbon and potentially earning carbon credits as additional commodities (Kuchment 2022). Given the continuing threat of erosion of arable topsoil in the United States (Du et al. 2022), high organic carbon deposition by the extensive root network of sorghum can contribute to critically needed management of this resource (Du et al. 2022; Ngidi et al. 2024). In summary, this system offers a promising avenue for reducing biofuel costs and improving soil quality, making biofuels a more feasible alternative to fossil fuels. This will also increase socioeconomic equity because pollution and climate change caused by fossil fuels disproportionately affect low-income people and people of color (Berberian et al. 2022; Ngcamu 2023).Figure 4.

## Conclusion

By reducing carbon emissions, second-generation biofuels, such as those made from sorghum, have the potential to lessen the disproportionate burdens vulnerable communities face (Economics of Biofuels; Jeswani et al. 2020; Levy & Patz 2015). The co-production of high-value terpenoids provides alternatives to carbon-intensive specialty chemicals, while tipping the scale towards economic feasibility of bioenergy crops. Through promoting biotechnological innovation mindful of broader societal and environmental impacts, this review strives to demonstrate the potential coupling bioproduct and biofuel generation can play in creating a more sustainable future.

## References

[CR1] Able JA, Rathus C, Godwin ID (2001) The investigation of optimal bombardment parameters for transient and stable transgene expression in Sorghum. Vitro Cell Development Biol Plant 37(3):341–348. 10.1007/s11627-001-0061-7

[CR2] Alieva NO, Konzen KA, Field SF, Meleshkevitch EA, Hunt ME, Beltran-Ramirez V, Miller DJ, Wiedenmann J, Salih A, Matz MV (2008) Diversity and evolution of coral fluorescent proteins. PLoS ONE 3(7):e2680. 10.1371/journal.pone.0002680 18648549 10.1371/journal.pone.0002680 PMC2481297

[CR3] Almeida LGFde, Parrella RAdaC, Simeone MLF, Ribeiro PCdeO, dos Santos AS, da Costa ASV, Guimarães AG, Schaffert RE (2019) Composition and growth of sorghum biomass genotypes for ethanol production. Biomass Bioenerg 122:343–348. 10.1016/j.biombioe.2019.01.030

[CR4] Alternative Fuel Price Report April 2024. (2024). https://afdc.energy.gov/files/u/publication/alternative_fuel_price_report_april_2024.pdf

[CR5] Ameen M, Mahmood A, Shahzad AN, Zia MA, Javaid MM (2024) Sorghum’s potential unleashed: a comprehensive exploration of bio-energy production strategies and innovations. Bioresour Technol Rep 27:101906. 10.1016/j.biteb.2024.101906

[CR6] Azodi CB, Lloyd JP, Shiu S-H (2020) The cis-regulatory codes of response to combined heat and drought stress in *Arabidopsis thaliana*. NAR Genomics Bioinform. 10.1093/nargab/lqaa049 10.1093/nargab/lqaa049 PMC7671360 33575601

[CR7] Barrero AF, Alvarez-Manzaneda EJ, Altarejos J, Salido S, Ramos JM (1993) Synthesis of Ambrox® from (−)-sclareol and (+)-cis-abienol. Tetrahedron 49(45):10405–10412. 10.1016/S0040-4020(01)80567-6

[CR8] Battraw M, Hall TC (1991) Stable transformation of *Sorghum bicolor* protoplasts with chimeric neomycin phosphotransferase II and β-glucuronidase genes. Theor Appl Genet 82(2):161–168. 10.1007/BF00226207 24213060 10.1007/BF00226207

[CR9] BCC Publishing. (2020). Global markets for flavors and fragrances. https://www-bccresearch-com.proxy1.cl.msu.edu/market-research/chemicals/flavors-fragrances-markets-report.html#

[CR10] Berberian AG, Gonzalez DJX, Cushing LJ (2022) Racial disparities in climate change-related health effects in the United States. Curr Environ Health Rep 9(3):451–464. 10.1007/s40572-022-00360-w 35633370 10.1007/s40572-022-00360-w PMC9363288

[CR11] Bibik JD, Sahu A, Kim B, Unda F, Andersen TB, Mansfield SD, Maravelias CT, Sharkey TD, Hamberger BR (2024) Engineered poplar for bioproduction of the triterpene squalene. Plant Biotechnol J 22(8):2301–2311. 10.1111/pbi.14345 38507185 10.1111/pbi.14345 PMC11258972

[CR12] Busta L, Schmitz E, Kosma DK, Schnable JC, Cahoon EB (2021) A co-opted steroid synthesis gene, maintained in sorghum but not maize, is associated with a divergence in leaf wax chemistry. Proc Natl Acad Sci U S A. 10.1073/pnas.2022982118 33723068 10.1073/pnas.2022982118 PMC8000359

[CR13] Cai X, Zhang X, Wang D (2011) Land availability for biofuel production. Environ Sci Technol 45(1):334–339. 10.1021/es103338e 21142000 10.1021/es103338e

[CR14] Casto AL, McKinley BA, Yu KMJ, Rooney WL, Mullet JE (2018) Sorghum stem aerenchyma formation is regulated by SbNAC_D during internode development. Plant Direct. 10.1002/pld3.85 31245693 10.1002/pld3.85 PMC6508845

[CR100] Chemelewski R, McKinley BA, Finlayson S, Mullet JE (2023) Epicuticular wax accumulation and regulation of wax pathway gene expression during bioenergy Sorghum stem development. Front Plant Sci. 14:1227859. 10.3389/fpls.2023.1227859 37936930 10.3389/fpls.2023.1227859 PMC10626490

[CR101] Cherwoo L, Gupta I, Flora G, Verma R, Kapil M, Arya SK, Ashokkumar V (2023) Biofuels an alternative to traditional fossil fuels: a comprehensive review. Sustain Energy Technol Assess. 60:103503. 10.1016/j.seta.2023.103503

[CR15] Dignac M-F, Derrien D, Barré P, Barot S, Cécillon L, Chenu C, Chevallier T, Freschet GT, Garnier P, Guenet B, Hedde M, Klumpp K, Lashermes G, Maron P-A, Nunan N, Roumet C, Basile-Doelsch I (2017) Increasing soil carbon storage: mechanisms, effects of agricultural practices and proxies. A review. Agron Sustain Dev 37(2):14. 10.1007/s13593-017-0421-2

[CR16] Du X, Jian J, Du C, Stewart RD (2022) Conservation management decreases surface runoff and soil erosion. Int Soil Water Conserv Res 10(2):188–196. 10.1016/j.iswcr.2021.08.001

[CR17] Duquesnoy E, Marongiu B, Castola V, Piras A, Porcedda S, Casanova J (2010) Combined analysis by GC (RI), GC-MS and ^13^C NMR of the supercritical fluid extract of *Abies alba* twigs. Nat Prod Commun 5(12):1995–1998 21299139

[CR18] Economics of biofuels. (n.d.). Retrieved August 19, 2024, from https://www.epa.gov/environmental-economics/economics-biofuels

[CR19] Elterlein F, Bugdahn N, Kraft P (2024) Sniffing out the sustainable future: the renewability revolution in fragrance chemistry. Chem Eur J. 10.1002/chem.202400006 38358844 10.1002/chem.202400006

[CR20] EnergyTech University Prize. (n.d.). Retrieved August 26, 2024, from https://www.energy.gov/technologytransitions/energytech-university-prize

[CR21] Farooq MA, Gao S, Hassan MA, Huang Z, Rasheed A, Hearne S, Prasanna B, Li X, Li H (2024) Artificial intelligence in plant breeding. Trends Genet 40(10):891–908. 10.1016/j.tig.2024.07.001 39117482 10.1016/j.tig.2024.07.001

[CR22] Fontanet-Manzaneque JB, Laibach N, Herrero-García I, Coleto-Alcudia V, Blasco-Escámez D, Zhang C, Orduña L, Alseekh S, Miller S, Bjarnholt N, Fernie AR, Matus JT, Caño-Delgado AI (2024) Untargeted mutagenesis of brassinosteroid receptor <scp>SbBRI1</scp> confers drought tolerance by altering phenylpropanoid metabolism in *Sorghum bicolor*. Plant Biotechnol J 22(12):3406–3423. 10.1111/pbi.14461 39325724 10.1111/pbi.14461 PMC11606431

[CR23] Franz v. Beiersdorf, Inc. et Al. (August 3, 2015).

[CR24] Fu J, McKinley B, James B, Chrisler W, Markillie LM, Gaffrey MJ, Mitchell HD, Orr G, Swaminathan K, Mullet J, Marshall-Colon A (2023) The stem cell-type transcriptome of bioenergy sorghum reveals the spatial regulation of secondary cell wall networks. BioRxiv. 10.1101/2023.04.22.53792138234846

[CR25] GM Crops List. (n.d.). Retrieved August 19, 2024, from https://www.isaaa.org/gmapprovaldatabase/cropslist/

[CR26] Grzonka, N. (2020). BASF and Isobionics launch Isobionics Santalol, an alternative to sandalwood oil. https://www.basf.com/global/en/media/news-releases/2020/07/p-20-244

[CR27] Hagio T, Blowers AD, Earle ED (1991) Stable transformation of sorghum cell cultures after bombardment with DNA-coated microprojectiles. Plant Cell Rep 10(5):260–264. 10.1007/BF00232571 24221592 10.1007/BF00232571

[CR28] Harfouche AL, Jacobson DA, Kainer D, Romero JC, Harfouche AH, Scarascia Mugnozza G, Moshelion M, Tuskan GA, Keurentjes JJB, Altman A (2019) Accelerating climate resilient plant breeding by applying next-generation artificial intelligence. Trends Biotechnol 37(11):1217–1235. 10.1016/j.tibtech.2019.05.007 31235329 10.1016/j.tibtech.2019.05.007

[CR29] Helmy M, Smith D, Selvarajoo K (2020) Systems biology approaches integrated with artificial intelligence for optimized metabolic engineering. Metab Eng Commun 11:e00149. 10.1016/j.mec.2020.e00149 33072513 10.1016/j.mec.2020.e00149 PMC7546651

[CR30] Herrera-Estrella L, Depicker A, Van Montagu M, Schell J (1983) Expression of chimaeric genes transferred into plant cells using a Ti-plasmid-derived vector. Nature 303(5914):209–213. 10.1038/303209a0 1422044

[CR31] Hossain MS, Islam MN, Rahman MM, Mostofa MG, Khan MAR (2022) Sorghum: a prospective crop for climatic vulnerability, food and nutritional security. J Agric Food Res 8:100300. 10.1016/j.jafr.2022.100300

[CR32] Iram A, Dong Y, Ignea C (2024) Synthetic biology advances towards a bio-based society in the era of artificial intelligence. Curr Opin Biotechnol 87:103143. 10.1016/j.copbio.2024.103143 38781699 10.1016/j.copbio.2024.103143

[CR33] Jeswani HK, Chilvers A, Azapagic A (2020) Environmental sustainability of biofuels: a review. Proc R Soc Lond A Math Phys Eng Sci. 10.1098/rspa.2020.0351 10.1098/rspa.2020.0351 PMC7735313 33363439

[CR34] Johnson SR, Bhat WW, Bibik J, Turmo A, Hamberger B, Hamberger B (2019) A database-driven approach identifies additional diterpene synthase activities in the mint family (Lamiaceae). J Biol Chem 294(4):1349–1362. 10.1074/jbc.RA118.006025 30498089 10.1074/jbc.RA118.006025 PMC6349103

[CR35] Kuchment, O. (2022). Bioenergy sorghum’s roots can replenish carbon in soil. https://bcbp.tamu.edu/department-updates/bioenergy-sorghums-roots-can-replenish-carbon-in-soil/

[CR36] Kyndt T, Quispe D, Zhai H, Jarret R, Ghislain M, Liu Q, Gheysen G, Kreuze JF (2015) The genome of cultivated sweet potato contains *Agrobacterium* T-DNAs with expressed genes: an example of a naturally transgenic food crop. Proc Natl Acad Sci U S A 112(18):5844–5849. 10.1073/pnas.1419685112 25902487 10.1073/pnas.1419685112 PMC4426443

[CR37] Lamb A, Weers B, McKinley B, Rooney W, Morgan C, Marshall-Colon A, Mullet J (2022) Bioenergy sorghum’s deep roots: a key to sustainable biomass production on annual cropland. GCB Bioenergy 14(2):132–156. 10.1111/gcbb.12907

[CR38] Leavell MD, McPhee DJ, Paddon CJ (2016) Developing fermentative terpenoid production for commercial usage. Curr Opin Biotechnol 37:114–119. 10.1016/j.copbio.2015.10.007 26723008 10.1016/j.copbio.2015.10.007

[CR39] Levy BS, Patz JA (2015) Climate change, human rights, and social justice. Ann Glob Health 81(3):310. 10.1016/j.aogh.2015.08.008 26615065 10.1016/j.aogh.2015.08.008

[CR40] Lin C-Y, Tian Y, Nelson-Vasilchik K, Hague J, Kakumanu R, Lee MY, Pidatala VR, Trinh J, De Ben CM, Dalton J, Northen TR, Baidoo EEK, Simmons BA, Gladden JM, Scown CD, Putnam DH, Kausch AP, Scheller HV, Eudes A (2022) Engineering sorghum for higher 4-hydroxybenzoic acid content. Metab Eng Commun 15:e00207. 10.1016/j.mec.2022.e00207 36188638 10.1016/j.mec.2022.e00207 PMC9519784

[CR41] Manus Bio Bioalternatives. (n.d.). Retrieved August 13, 2024, from https://www.manusbio.com/bioalternatives

[CR42] MarketsGlob. (2024). Global Ambroxide Market 2024 - Industry analysis by player, region, type, application and sales channel, forecast. https://marketsglob.com/report/ambroxide-market/4838/

[CR43] McCormick RF, Truong SK, Sreedasyam A, Jenkins J, Shu S, Sims D, Kennedy M, Amirebrahimi M, Weers BD, McKinley B, Mattison A, Morishige DT, Grimwood J, Schmutz J, Mullet JE (2018) The *Sorghum bicolor* reference genome: improved assembly, gene annotations, a transcriptome atlas, and signatures of genome organization. Plant J 93(2):338–354. 10.1111/tpj.13781 29161754 10.1111/tpj.13781

[CR44] Meki MN, Snider JL, Kiniry JR, Raper RL, Rocateli AC (2013) Energy sorghum biomass harvest thresholds and tillage effects on soil organic carbon and bulk density. Ind Crops Prod 43:172–182. 10.1016/j.indcrop.2012.07.033

[CR45] Miller S, Rønager A, Holm R, Fontanet-Manzaneque JB, Caño-Delgado AI, Bjarnholt N (2023) New methods for sorghum transformation in temperate climates. AoB Plants. 10.1093/aobpla/plad030 37396498 10.1093/aobpla/plad030 PMC10308921

[CR46] Mo GCH, Posner C, Rodriguez EA, Sun T, Zhang J (2020) A rationally enhanced red fluorescent protein expands the utility of FRET biosensors. Nat Commun 11(1):1848. 10.1038/s41467-020-15687-x 32296061 10.1038/s41467-020-15687-x PMC7160135

[CR47] Montesantos N, Maschietti M (2020) Supercritical carbon dioxide extraction of lignocellulosic bio-oils: the potential of fuel upgrading and chemical recovery. Energies 13(7):1600. 10.3390/en13071600

[CR48] Moose SP, Sisco PH (1996) Glossy15, an APETALA2-like gene from maize that regulates leaf epidermal cell identity. Genes Dev 10(23):3018–3027. 10.1101/gad.10.23.3018 8957002 10.1101/gad.10.23.3018

[CR49] Naraian, R., & Gautam, R. L. (2018). Penicillium enzymes for the saccharification of lignocellulosic feedstocks. In *New and future developments in microbial biotechnology and bioengineering*, Elsevier, Amsterdam, pp. 121–136

[CR50] National Transportation Safety Board. (2018). Derailment and hazardous materials release of union pacific railroad unit ethanol train graettinger, Iowa. https://www.ntsb.gov/investigations/accidentreports/reports/rar1802.pdf

[CR51] Ng RTL, Maravelias CT (2017) Design of biofuel supply chains with variable regional depot and biorefinery locations. Renew Energy 100:90–102. 10.1016/j.renene.2016.05.009

[CR52] Ngcamu BS (2023) Climate change effects on vulnerable populations in the global south: a systematic review. Nat Hazards 118(2):977–991. 10.1007/s11069-023-06070-2

[CR53] Ngidi A, Shimelis H, Abady S, Figlan S, Chaplot V (2024) Response of *Sorghum bicolor* genotypes for yield and yield components and organic carbon storage in the shoot and root systems. Sci Rep 14(1):9499. 10.1038/s41598-024-59956-x 38664438 10.1038/s41598-024-59956-x PMC11045799

[CR55] Parrott, W., Assmann, S., Gordon-Kamm, W., Schmutz, J., Veena, V., & Young, M. (2024). Overcoming barriers in plant transformation: a focus on bioenergy crops10.2172/2335710

[CR56] Perfumer’s World. (n.d.). *Ambrofix*. Retrieved October 21, 2024, from https://www.perfumersworld.com/view.php?pro_id=1UW16861#Docs

[CR57] Polaris Market Research. (2024). Squalene market share, size, trends, industry analysis report, by source (Animal, Amaranth Oil, Synthetic); By End-Use; By Region; Segment Forecast, 2024–2032. https://www.polarismarketresearch.com/industry-analysis/squalene-market#:~:text=Report%20Outlook,10.5%25%20during%20the%20forecast%20period.

[CR58] Popa O, Băbeanu NE, Popa I, Niță S, Dinu-Pârvu CE (2015) Methods for obtaining and determination of squalene from natural sources. BioMed Res Int 2015:1–16. 10.1155/2015/367202 10.1155/2015/367202 PMC4324104 25695064

[CR59] Presley DR, Sindelar AJ, Buckley ME, Mengel DB (2012) Long-term nitrogen and tillage effects on soil physical properties under continuous grain sorghum. Agron J 104(3):749–755. 10.2134/agronj2011.0311

[CR60] Rai KK (2022) Integrating speed breeding with artificial intelligence for developing climate-smart crops. Mol Biol Rep 49(12):11385–11402. 10.1007/s11033-022-07769-4 35941420 10.1007/s11033-022-07769-4 PMC9360691

[CR61] Ranaweera T, Brown BNI, Wang P, Shiu S-H (2022) Temporal regulation of cold transcriptional response in switchgrass. Front Plant Sci. 10.3389/fpls.2022.998400 36299783 10.3389/fpls.2022.998400 PMC9589291

[CR62] Reed J, Stephenson MJ, Miettinen K, Brouwer B, Leveau A, Brett P, Goss RJM, Goossens A, O’Connell MA, Osbourn A (2017) A translational synthetic biology platform for rapid access to gram-scale quantities of novel drug-like molecules. Metab Eng 42:185–193. 10.1016/j.ymben.2017.06.012 28687337 10.1016/j.ymben.2017.06.012 PMC5555447

[CR63] Rushton PJ, Torres JT, Parniske M, Wernert P, Hahlbrock K, Somssich IE (1996) Interaction of elicitor-induced DNA-binding proteins with elicitor response elements in the promoters of parsley PR1 genes. EMBO J 15(20):5690–5700. 10.1002/j.1460-2075.1996.tb00953.x 8896462 PMC452313

[CR64] Sainju UM, Singh HP, Singh BP (2015) Cover crop effects on soil carbon and nitrogen under bioenergy sorghum crops. J Soil Water Conserv 70(6):410–417. 10.2489/jswc.70.6.410

[CR65] Schumaker B, Mortensen L, Klein RR, Mandal S, Dykes L, Gladman N, Rooney WL, Burson B, Klein PE (2024) UV-induced reactive oxygen species and transcriptional control of 3-deoxyanthocyanidin biosynthesis in black sorghum pericarp. Front Plant Sci. 10.3389/fpls.2024.1451215 39435026 10.3389/fpls.2024.1451215 PMC11491397

[CR66] Sharma R, Liang Y, Lee MY, Pidatala VR, Mortimer JC, Scheller HV (2020) *Agrobacterium*-mediated transient transformation of sorghum leaves for accelerating functional genomics and genome editing studies. BMC Res Notes 13(1):116. 10.1186/s13104-020-04968-9 32103777 10.1186/s13104-020-04968-9 PMC7045639

[CR67] Shi P, Fu X, Liu M, Shen Q, Jiang W, Li L, Sun X, Tang K (2017) Promotion of artemisinin content in *Artemisia annua* by overexpression of multiple artemisinin biosynthetic pathway genes. Plant Cell, Tissue and Organ Culture (PCTOC) 129(2):251–259. 10.1007/s11240-017-1173-z

[CR68] Silva TN, Thomas JB, Dahlberg J, Rhee SY, Mortimer JC (2022) Progress and challenges in sorghum biotechnology, a multipurpose feedstock for the bioeconomy. J Exp Bot 73(3):646–664. 10.1093/jxb/erab450 34644381 10.1093/jxb/erab450 PMC8793871

[CR69] Stamenković OS, Siliveru K, Veljković VB, Banković-Ilić IB, Tasić MB, Ciampitti IA, Đalović IG, Mitrović PM, Sikora VŠ, Prasad PVV (2020) Production of biofuels from sorghum. Renew Sustain Energy Rev 124:109769. 10.1016/j.rser.2020.109769

[CR70] Standard operating procedure (SOP) Template for APHIS BRS plant permits. (n.d.). Retrieved August 26, 2024, from https://www.aphis.usda.gov/sites/default/files/sop-template-for-aphis-brs-plant-permits.pdf

[CR71] Stockmann U, Adams MA, Crawford JW, Field DJ, Henakaarchchi N, Jenkins M, Minasny B, McBratney AB, Courcelles VdeRde, Singh K, Wheeler I, Abbott L, Angers DA, Baldock J, Bird M, Brookes PC, Chenu C, Jastrow JD, Lal R, Zimmermann M (2013) The knowns, known unknowns and unknowns of sequestration of soil organic carbon. Agric Ecosyst Environ 164:80–99. 10.1016/j.agee.2012.10.001

[CR72] U.S. Department of Energy. (n.d.). Biodiesel Laws and Incentives in Federal. Retrieved October 28, 2024, from https://afdc.energy.gov/fuels/laws/BIOD?state=US#:~:text=Advanced%20Biofuel%20Feedstock%20Incentives&text=Qualified%20feedstock%20producers%20are%20eligible,15%20years%20for%20woody%20feedstocks.

[CR73] U.S. Energy Information Administration. (2024). U.S. energy facts explained. https://www.eia.gov/energyexplained/us-energy-facts/

[CR74] USC Ch. 9: federal food, drug, and cosmetic act (1938). https://uscode.house.gov/view.xhtml?path=/prelim@title21/chapter9&edition=prelim

[CR75] Uludere Aragon NZ, Parker NC, VanLoocke A, Bagley J, Wang M, Georgescu M (2022) Sustainable land use and viability of biojet fuels. Nat Sustain 6(2):158–168. 10.1038/s41893-022-00990-w

[CR76] University of Wisconsin–Madison department of pathology and laboratory medicine. (n.d.). *IHC & ISH*. Retrieved November 3, 2024, from https://research.pathology.wisc.edu/ihc-ish/

[CR77] USDA Production - Sorghum. (n.d.). Retrieved August 18, 2024, from https://fas.usda.gov/data/production/commodity/0459200

[CR78] Uwineza PA, Waśkiewicz A (2020) Recent advances in supercritical fluid extraction of natural bioactive compounds from natural plant materials. Molecules 25(17):3847. 10.3390/molecules25173847 32847101 10.3390/molecules25173847 PMC7504334

[CR79] Winchell F, Stevens CJ, Murphy C, Champion L, Fuller DQ (2017) Evidence for sorghum domestication in fourth millennium BC Eastern Sudan: spikelet morphology from ceramic impressions of the Butan group. Curr Anthropol 58(5):673–683. 10.1086/693898

[CR80] Wisconsin crop innovation center homepage. (n.d.). Retrieved August 19, 2024, from https://cropinnovation.cals.wisc.edu/

[CR81] Xiao Y, Li X, Yao L, Xu D, Li Y, Zhang X, Li Z, Xiao Q, Ni Y, Guo Y (2020) Chemical profiles of cuticular waxes on various organs of *Sorghum bicolor* and their antifungal activities. Plant Physiol Biochem 155:596–604. 10.1016/j.plaphy.2020.08.026 32846395 10.1016/j.plaphy.2020.08.026

[CR82] Xu Y, Liu X, Cao X, Huang C, Liu E, Qian S, Liu X, Wu Y, Dong F, Qiu C-W, Qiu J, Hua K, Su W, Wu J, Xu H, Han Y, Fu C, Yin Z, Liu M, Roepman R, Dietmann S, Virta M, Kengara F, Zhang Ze, Zhang L, Zhao T, Dai Ji, Yang J, Lan L, Luo M, Liu Z, An T, Zhang B, He X, Cong S, Zhang W, Lewis JP, Tiedje JM, Wang Qi, An Z, Wang F, Huang T, Lu C, Cai Z, Zhang J (2021) Artificial intelligence: a powerful paradigm for scientific research. Innovation (Camb) 2(4):100179. 10.1016/j.xinn.2021.100179 34877560 10.1016/j.xinn.2021.100179 PMC8633405

[CR83] Yang M, Baral NR, Simmons BA, Mortimer JC, Shih PM, Scown CD (2020) Accumulation of high-value bioproducts in planta can improve the economics of advanced biofuels. Proc Natl Acad Sci U S A 117(15):8639–8648. 10.1073/pnas.2000053117 32220956 10.1073/pnas.2000053117 PMC7165473

[CR84] Zhang P, Li S-T, Liu T-T, Fu C-H, Zhou P-P, Zhao C-F, Yu L-J (2011) Overexpression of a 10-deacetylbaccatin III-10 β-O-acetyltransferase gene leads to increased taxol yield in cells of *Taxus chinensis*. Plant Cell Tissue Organ Cult (PCTOC) 106(1):63–70. 10.1007/s11240-010-9894-2

[CR85] Zhao ZY, Cai T, Tagliani L, Miller M, Wang N, Pang H, Rudert M, Schroeder S, Hondred D, Seltzer J, Pierce D (2000) Agrobacterium-mediated sorghum transformation. Plant Mol Biol 44(6):789–798 11202440 10.1023/a:1026507517182

[CR86] Zhou C, Yuan Z, Ma X, Yang H, Wang P, Zheng L, Zhang Y, Liu X (2021) Accessible chromatin regions and their functional interrelations with gene transcription and epigenetic modifications in sorghum genome. Plant Commun 2(1):100140. 10.1016/j.xplc.2020.100140 33511349 10.1016/j.xplc.2020.100140 PMC7816095

